# Motorik-Module (MoMo) – the KiGGS Wave 2 module to survey motor performance and physical activity

**DOI:** 10.17886/RKI-GBE-2017-110

**Published:** 2017-09-27

**Authors:** Alexander Woll, Claudia Albrecht, Annette Worth

**Affiliations:** 1 Karlsruhe Institute of Technology, Institute of Sports and Sports Science; 2 University of Education Karlsruhe, Institute of Physical Education and Sports

**Keywords:** MOTOR PERFORMANCE, PHYSICAL ACTIVITY, ACCELEROMETRY, HEALTH MONITORING, KIGGS

## Abstract

Initially, the Motorik-Module (MoMo) Longitudinal Study was surveyed between 2003 and 2006 using a sub-sample from the baseline German Health Interview and Examination Survey for Children and Adolescents (KiGGS). The federal representative sub-sample of KiGGS consisted of 4,528 children and adolescents aged 4 to 17. To date, there have been two further survey waves: 2009-2012 (Wave 1) and 2015-2017 (Wave 2). MoMo Wave 2 consists of motor performance tests, anthropometric measurements, questionnaire-based information collection on activity habits and motion sensor data. Initial results of MoMo Wave 2 will be published in the second half of 2018. The total size of the Wave 2 sample is estimated to reach 5,200 participants. As its central goal, the MoMo longitudinal survey aims to contribute towards the long-term improvement of child and adolescent health in Germany. A focus will be on developmental (age-related) and periodic (over time) trends in motor performance and physical activity, underlying factors and the impacts on physical and mental health in the development of children and adolescents.

## 1. Background and objective

Surveying motor performance and physical activity patterns in the development and health of children and adolescents is essential and plays a particularly important role in health promotion [1-3]. As is well-known, regular physical exercise benefits health and helps prevent diseases; good motor performance is also a protective factor for the cardiovascular system [4-6]. Inversely, experts blame the increasingly sedentary lifestyle of children, adolescents and adults for the progress of diseases such as obesity [7], cardiovascular diseases [2] and mental disorders [8]. In adults, this connection between physical activity behaviour, motor performance and health has been widely established [9]; however, there are still gaps in research involving children and adolescents [8, 10].

The MoMo (Motorik-Module) survey has been a module of the representative German Health Interview and Examination Survey for Children and Adolescents (KiGGS) of the Robert Koch Institute since the 2003-2006 baseline study [11]. MoMo collects data on motor performance, anthropometric measurements as well as levels of physical activity among children and adolescents. The MoMo module in particular aims to analyse a) the developmental (age-related) and periodic (over time) trends of motor performance and physical activity as well as b) the underlying factors and c) the effects of motor performance and physical activity on the health development of children and adolescents. The MoMo module provides supplementary in-depth information on motor performance and physical activity for a sub-sample of KiGGS study respondents. Linking the data collected by the MoMo and KiGGS studies offers the unique opportunity to combine the representative physical and mental health and health behaviour data of children and adolescents in Germany with detailed data on physical activity and motor performance.

**Motorik-Module Wave 2**

Third time of data collection for the Motorik-Module Study Physical fitness and physical activity as determinants of health development in children and adolescents, 2015-2017

**Acronym: MoMo** - **Mo**torik-**Mo**dule-Study

**Implementation:** Karlsruhe Institute of Technology, Institute of Sports and Sports Science

**Aim:** Providing reliable data on motor performance and physical activity among children and adolescents in Germany as a basis to analyse trends and for longitudinal analyses as well the analysis of links between motor performance and levels of physical activity on the one hand and health development among children and adolescents on the other.

**Study design**: Combined cross-sectional and cohort examination and interview survey

**MoMo cross-sectional study**

**Population:** Children and adolescents with permanent residence in Germany

**Sampling:** MoMo Wave 2 participants are randomly sampled from the cross-sectional sample of KiGGS Wave 2 (registry office sample). Prior participation in KiGGS Wave 2 is prerequisite for an invitation to MoMo Wave 2.

**Age range:** 4-17 years

**Sample size:** Approximately 5.200 participants (expected)

**MoMo cohort study**

**Sampling:** Renewed invitation of all participants in the MoMo baseline survey (2003-2006; then aged 0-17) or MoMo Wave 1 (2009-2012) willing to take part again. Prior participation in KiGGS Wave 2 is prerequisite for an invitation to MoMo Wave 2.

**Age range:** 10-29 years

**Sample size:** Approximately 3,000 returning participants

**Survey period:** January 2015-September 2017

More Information in German is available at www.motorik-modul.de

Based on representative data for the German population (as of 2004), the MoMo baseline survey (2003-2006, 167 sample points, n=4,528) delivered age- and gender-specific norm values of motor performance of children and adolescents aged 4 to 17, as well as data collection on activity levels based on a standardised method [12]. In order to record over time the development of motor performance and physical activity levels of children and adolescents, MoMo Wave 1 (2009-2012) and Wave 2 (2015-2017) invited the children, adolescents and young adults who had participated in the baseline survey for renewed examinations. The third survey wave is scheduled for 2018-2020. Continuing the MoMo survey, based on a cohort-sequential design, will for the first time make it possible to combine reliable cohort comparisons [13], as well as longitudinal analyses of motor performance and physical activity and the health of children and adolescents aged 4 to 28 (as of MoMo Wave 2) [11, 14, 15]. Cross-sectional analyses of different samples in the form of cohort comparisons performed at different time points can help researchers trace changes across the total sample. In longitudinal analyses, in contrast, the same survey participants are examined and questioned at different time points. These analyses can reveal changes for a single individual and highlight causal relations between different variables.

Currently the MoMo module is in the data collection phase for Wave 2. The following sections address this phase and the methodology applied.

## 2. Methodology

### 2.1 Study design and sampling

The MoMo module consists of both a cross-sectional and a longitudinal component. The cross-sectional sample of the MoMo survey comprises a sub-sample of participants from the cross-sectional sample of KiGGS Wave 2 aged 4 to 17. The article New data for action. Data collection for KiGGS Wave 2 has been completed in this issue of the Journal of Health Monitoring contains a detailed description of the target population and sampling for the KiGGS study. Children and adolescents were randomly drawn from the gross sample and assigned to the MoMo module during sampling for KiGGS Wave 2 independently of their participation in KiGGS. Participation in KiGGS Wave 2 was a prerequisite for an invitation to participate in the MoMo survey.

**Figure 1:**
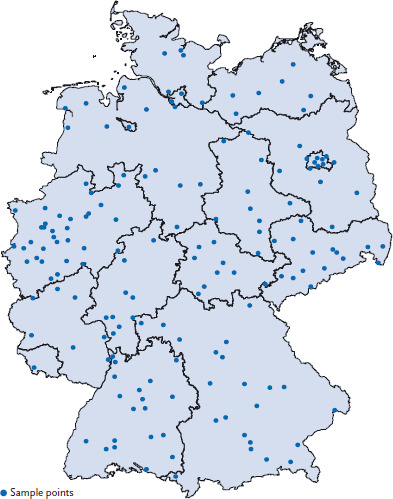
Sample points of MoMo Wave 2 Source: RKI

The longitudinal sample for the MoMo module was comprised of MoMo baseline survey (2003-2006) and MoMo Wave 1 (2009-2012) respondents that also participated in KiGGS Wave 2. Participants examined and interviewed in the longitudinal survey were therefore aged up to 23 in MoMo Wave 1 and up to 29 in MoMo Wave 2. After KiGGS respondents agreed to participate in MoMo, MoMo respondents from both the cross-sectional and longitudinal samples were invited to one of the 167 selected sample points (Figure 1) for motor performance tests, anthropometric measurements and to record data on their physical activity.

Data collection for Wave 2 of the MoMo survey began in January 2015 and will end in September 2017. Until May 2017, 56 routes were completed; around 4,000 participants were tested and interviewed. Further thirty sample points will be visited by the end of September 2017; the total number of participants in Wave 2 is estimated to reach 5,200 respondents.

Germany’s federal commissioner for data protection has been informed of and has approved the survey. A number of technical and organisational measures (including control of data carriers, data storage, users, data access and data transmission) detailed in a comprehensive procedure protocol, ensure compliance with data protection regulations. All of the data collected during the survey is pseudonymised. Participation in the survey is voluntary. Participants, and where necessary their parents or legal guardians, are informed about the goals and content of the survey as well as the measures taken to protect their data, to be able to give their informed consent. The study (funding code 01ER1503) received a positive vote from the ethics commission of the Karlsruhe Institute of Technology on 23 September 2014.

### 2.2 Assessment methods and testing instruments

Based on the survey’s longitudinal approach, the instruments to collect data in Wave 2 are largely the same as those used in the baseline study and Wave 1. MoMo longitudinal survey instruments include a test profile to survey motor performance, anthropometric measurements, a questionnaire to collect data on physical activity, as well as use of a motion sensor (accelerometer).

The development and basis of the MoMo motor performance test profile and the MoMo physical activity questionnaire have been extensively published and documented in test manuals [16-18]. The quality of the motor performance tests and questionnaire has been evaluated in pre-tests and supplementary studies [12, 18].

The test setup, instructions and testing itself were conducted in accordance with the instructions contained in the test manual and have already been described [16]. The test, including the filling-in of the questionnaire, takes 70-90 minutes to complete. The test teams consist of four to six trained test instructors from the Institute of Sports and Sports Science (IFSS) at the Karlsruhe Institute of Technology.

#### Motor performance testing and anthropometric measurements

The motor performance test profile comprises eleven tasks (Table 1). Motor performance test items were selected to suit children aged 4 to 17 and with slight modifications, adults as well.

Anthropometric measurements included height and weight, waist circumference, data on body composition (body fat percentage, body cell mass via bioelectrical impedance analysis, BIA) and blood pressure at rest. Trained survey staff used standardised and quality-controlled procedures [16].

Technological developments and new findings published in the course of the MoMo survey waves made necessary minimal changes to test tools. For Wave 2, the individual body weight-related cycling endurance protocols used in the baseline study and Wave 1 were adapted to conform to comparable protocols of the World Health Organization [19]. Whereas Wave 1 used four-channel BIA measurement devices, Wave 2 switched to multi-frequency eight-channel measurements by body segment.

**KiGGS Wave 2**

Second follow-up to the German Health Interview and Examination Survey for Children and Adolescents

**Data owner:** Robert Koch Institute

**Aim:** Providing reliable information on health status, health-related behaviour, living conditions, protective and risk factors, and health care among children, adolescents and young adults living in Germany, with the possibility of trend and longitudinal analyses.

**Study design**: Combined cross-sectional and cohort study conducted as an examination and interview survey

**KiGGS cross-sectional study**

**Population:** Children and adolescents with permanent residence in Germany

**Sampling:** Samples from official residency registries - randomly selected children and adolescents from the 167 cities and municipalities covered by the KiGGS baseline study

**Age range:** 0-17 years

**Sample size:** Approximately 15,000 participants

**KiGGS cohort study**

**Sampling:** Re-invitation of everyone who took part in the KiGGS baseline study (2003-2006; aged between 0 and 17 at that time) and who was willing to participate in a follow-up

**Age range:** 10-29 years

**Sample size:** Approximately 10,000 follow-up participants

**Survey period:** September 2014-August 2017

**Modules:** BELLA, EsKiMo, GerES, KiESEL, MoMo

More information is available at www.kiggs-studie.de/english

#### MoMo physical activity questionnaire

The standardised MoMo physical activity questionnaire (MoMo-AFB) takes into account the different life stages of respondents, so that different versions are available for nursery school and school children as well as for adolescents and young adults [12, 17]. Participants in the MoMo survey aged 11 and older fill out the MoMo-AFB by themselves. 4-to 10-year-olds are interviewed, with their parents or guardians filling out the questionnaire. The MoMo-AFB covers various fields of physical activity (active commutes, everyday physical activity, physical activity during leisure time, physical activity at school or at work). The intensity, type, frequency and duration of such physical activity and seasonal changes in activity levels are also taken into account. Psychosocial and environmental factors influencing physical activity are also recorded.

#### Motion sensor data

Since Wave 2, the MoMo physical activity questionnaire has been supplemented by data on physical activity recorded objectively by means of a motion sensor (accelerometer). The survey uses the Actigraph GT3X+/wGT3X-BT accelerometer. These accelerometers are internationally recognised and the de facto scientific standard for measuring physical activity [20]. Participants in the MoMo module aged 6 and older are instructed to wear their accelerometer on eight consecutive days during the daytime. To quantify physical activity, the accelerometer is worn above the hip on the right side of the body. Currently (as of May 2017), data sets for 1,898 respondents aged 6 to 20 have been collected.

## 3. Discussion and outlook

The MoMo survey is among the few – in German-speaking countries the only – population-based longitudinal survey of motor performance and physical activity. Data from Wave 2 (2015-2017) will provide answers to complex questions on the developments of physical activity/inactivity and motor performance among children, adolescents and young adults in Germany, as well as on the factors influencing these developments. Data from three distinct time points enable researchers to conduct complex statistical calculations that can, for example, help model non-linear developments, for instance development curves when growth is limited, as well as the calculation of interdependencies between different factors influencing these developments. This provides the necessary basis for an assessment of the effects of physical activity and motor performance on both the objective and subjective parameters of physical and mental health. The further development of methods in Wave 2, such as the first-time use of accelerometers, promise to pave the way for more differentiated analyses.

The expected results of Wave 2 provide the basis for important health policy recommendations at numerous levels. Health policy can use the knowledge available on the factors influencing physical activity and motor performance levels and their health-relatedness to identify those people potentially at risk early on and provide them with targeted support. The long-term and regular monitoring of motor performance and physical activity creates a reliable database to assess the effectivity of initiatives that have been promoting sports activities over the past years in Germany.

Initial results from MoMo Wave 2 will be made available to the interested public in the second quarter of 2018. In parallel to the evaluation phase of Wave 2, which is set to begin in September 2017, preparations for Wave 3 (2018-2020) data collection will begin.

## Key statements

- MoMo (Motorik-Module Study) has been a module of the KiGGS study since the 2003-2006 baseline study.
- MoMo Wave 2 data collection began in January 2015 and will end in September 2017.
- MoMo surveys motor performance and physical activity.
- MoMo Wave 3 starts in 2018.

## Figures and Tables

**Table 1:** MoMo motor performance test items Source: Modified according to [16]

Test item	Baseline study	Wave 1	Wave 2	Test item	Baseline study	Wave 1	Wave 2
Reaction time	◆ (4-10)		◆ (4-10)	Push-ups			
	○ (11-17)	○ (4-23)	○ (4-28)		○ (6-17)	○ (6-23)	○ (6-28)
Line tracking	◆ (4-10)			Jumping on a force plate			
	○ (11-17)	○ (4-23)	○ (4-28)		○ (4-17)	○ (4-23)	○ (4-28)
Inserting pins	◆ (4-10)			Jumping-sideways	◆ (4-10)		◆ (4-10)
	○ (11-17)	○ (4-23)	○ (4-28)		○ (11-17)	○ (4-23)	○ (4-28)
Static Stand	◆ (4-10)		◆ (4-10)	Sit-ups			
	○ (11-17)	○ (4-23)	○ (4-28)		–	○ (6-23)	○ (6-28)
Balancing backwards				Stand & reach	◆ (4-10)		◆ (4-10)
	○ (4-17)	○ (4-23)	○ (4-28)		○ (11-17)	○ (4-23)	○ (4-28)
Standing long jump				Ergometric endurance testing	◆ (11-17)		○* (6-28)
	○ (4-17)	○ (4-23)	○ (4-28)		○ (6-10)	○ (6-23)	◆* (11-23)
							including lactate test

◆ KiGGS

○ MoMo

* Switch from related to bodyweight ergometer protocol to the protocol of the World Health Organization (after age 10, starts with 25 watts, with load increases of 25 watts every two minutes)
